# First documented cases of equine leishmaniasis caused by *Leishmania* (*Mundinia*) *martiniquensis* in France

**DOI:** 10.1051/parasite/2026054

**Published:** 2026-09-28

**Authors:** Charline Alleaume, Eve Laloy, Marie-Capucine Dupuis-Tricaud, Nada Kuk, Emilie Kariya, Nicolas Keck, Jérome Depaquit, Laurence Lachaud, Christophe Ravel

**Affiliations:** 1 Vetodiag Laboratory 14170 Saint-Pierre en Auge France; 2 University of Montpellier & UMR5290 IRD MiVEGEC 34090 Montpellier France; 3 University of Reims Champagne-Ardenne, UR ESCAPE-USC ANSES PETARD 51100 Reims France & Laboratoire de biologie territoriale, CHU de Reims 51100 Reims France; 4 Departmental Veterinary Laboratory of Hérault 34000 Montpellier France; 5 French Reference Centre for Leishmaniasis 34090 Montpellier France

**Keywords:** *Leishmania martiniquensis*, Equine leishmaniasis, *Equus caballus*, Cutaneous leishmaniasis, *Mundinia*, France

## Abstract

In this study, we describe the first documented cases of equine cutaneous leishmaniasis caused by *Leishmania* (*Mundinia*) *martiniquensis* in France and provide clinical and parasitological data relevant for the recognition of this emerging infection in horses. Three horses presenting localized cutaneous lesions were diagnosed with *L. martiniquensis* infection in different regions of France without spatial or temporal clustering. Lesions were clinically nonspecific and initially compatible with common equine dermatological conditions, including sarcoid-like tumors. Histopathological examination revealed granulomatous dermatitis with intrahistiocytic organisms morphologically consistent with *Leishmania* amastigotes in all cases. Molecular analysis of formalin-fixed paraffin-embedded tissue confirmed the presence of *Leishmania* DNA, and sequencing of the ITS1 locus identified *L. martiniquensis*. Direct agglutination test serology was negative in two horses and strongly positive in one. In one case, parasite isolation was achieved in culture. The disease remained localized in all animals, with spontaneous regression in one case, resolution following surgical excision in another, and complete healing after intralesional treatment with meglumine antimoniate in the third.

## Abstract (fr)

Cette étude décrit les premiers cas documentés de leishmaniose cutanée équine due à *Leishmania (Mundinia) martiniquensis* en France et fournit des données cliniques et parasitologiques utiles pour identifier cette infection émergente chez les chevaux. Trois chevaux présentant des lésions cutanées localisées ont reçu un diagnostic d'infection à *L. martiniquensis* dans différentes régions de France, sans regroupement spatial ou temporel. Les lésions étaient cliniquement aspécifiques et initialement compatibles avec des affections dermatologiques équines courantes, notamment des tumeurs de type sarcoïde. L'examen histopathologique a révélé, dans tous les cas, une dermatite granulomateuse avec des organismes intrahistiocytaires morphologiquement compatibles avec des amastigotes de *Leishmania*. L'analyse moléculaire de tissus fixés au formol et inclus en paraffine a confirmé la présence d'ADN de *Leishmania*, et le séquençage du locus ITS1 a permis d'identifier *L. martiniquensis*. La sérologie par test d'agglutination directe s'est révélée négative chez deux chevaux et fortement positive chez un troisième. Dans un cas, l'isolement du parasite a été obtenu en culture. La maladie est restée localisée chez tous les animaux, avec une régression spontanée dans un cas, une résolution après exérèse chirurgicale dans un autre, et une guérison complète suite à un traitement intralésionnel par l'antimoniate de méglumine.

## Introduction

Leishmaniases are protozoan parasitic diseases affecting humans and a wide range of domestic and wild animal species. Transmission of *Leishmania* parasites is primarily vector-borne, through the bite of infected female phlebotomine sand flies, mainly species of the genera *Phlebotomus* in the Old World and *Lutzomyia* sensu lato in the New World. In Europe, *Leishmania infantum* is the endemic species and the main etiological agent of human and canine leishmaniasis, with dogs acting as the main domestic reservoir [4]. In equids living in endemic areas, infection with *L. infantum* is well documented and is characterized by frequent exposure. Despite high seroprevalence, infections are predominantly asymptomatic, and clinical cases are rarely reported [1, 3, 7, 11].

By contrast, *Leishmania martiniquensis* Desbois, Pratlong & Dedet, 2014 belongs to a distinct phylogenetic lineage for which the ecology and epidemiology remain poorly characterized [5]. The vector ecology of species belonging to the subgenus *Mundinia* remains incompletely resolved. However, experimental transmission studies and observations under natural conditions have implicated biting midges of the genus *Culicoides* as potential vectors [2, 8]. *Leishmania martiniquensis* was initially described from human cases in the Caribbean and has subsequently been reported in Southeast Asia and sporadically in South America [5, 9, 15]. Unlike for *L. infantum*, limited data are currently available on the prevalence or clinical expression of *L. martiniquensis* infection in equids.

Over the last decade, fewer than 20 equine infections attributed to *L. martiniquensis* have been reported worldwide, mainly in Europe and the Americas [12–14]. These cases were typically associated with localized and poorly specific cutaneous lesions. A case of bovine leishmaniasis has also been reported in Switzerland [10]. Their rarity, wide geographic dispersion, and temporal spread do not support the identification of a structured epidemiologic pattern. However, they indicate that this species should be considered in the differential diagnosis of equine cutaneous lesions, including outside areas traditionally recognized as endemic for leishmaniasis.

To date, no equine infections with *L. martiniquensis* have been reported in France. The present study describes three equine cases diagnosed in different regions of the country, without any spatial or temporal clustering. These observations represent the first documented report of equine *L. martiniquensis* infection in France and we provide descriptive clinical and parasitological data relevant for future regional investigations.

## Materials and methods

### Ethics

All procedures were performed as part of routine diagnostic investigations with informed consent from the animal owners. No experimental procedures were conducted specifically for research purposes.

### Cases and samples

Three horses presenting localized cutaneous lesions were investigated in France as part of routine diagnostic procedures. Depending on the case, skin biopsy specimens or surgically excised lesion samples were obtained and submitted for histopathological and molecular analyses. Formalin-fixed, paraffin-embedded (FFPE) tissue was available for all three cases. In one case, fresh lesion material was also available for parasite isolation.

### Histopathology

Tissue samples were processed routinely for histopathological examination. Sections were stained with hematoxylin–eosin–saffron and examined by light microscopy.

### DNA extraction, molecular detection, and species identification

DNA was extracted from FFPE tissue using a QIAamp DNA FFPE Tissue Kit (QIAGEN, Hilden, Germany), according to the manufacturer’s instructions. Molecular detection of *Leishmania* DNA was performed by PCR amplification of the internal transcribed spacer 1 (ITS1) using primers LITSR and L5.8S, originally described by el Tai et al. [6]. A 348-bp amplicon was obtained from each case and species identification was achieved by bidirectional sequencing of the PCR products. The sequences obtained from the three cases identified the parasite as *Leishmania martiniquensis*. The new sequences have been deposited in GenBank under accession numbers PX959956–PX959958.

### Serology

Serological testing was performed using an in-house direct agglutination test (DAT), originally developed for canine leishmaniasis, using *Leishmania infantum* strain MCAN/FR/88/RM2 as the antigen source. Serum titers ≥1:200 were considered positive. The diagnostic sensitivity and specificity of this assay have not been established in horses, and its performance for detecting *L. martiniquensis* infection in equids has not been formally evaluated.

### Parasite isolation

In one case, parasite isolation was achieved in culture from lesion material in M199 medium supplemented with 10% heat-inactivated fetal calf serum and incubated at 26 °C. The identity of the isolate was confirmed by ITS1 sequencing; the sequence obtained from the cultured isolate was identical to that obtained directly from the corresponding tissue sample (Case 3, GenBank accession number PX959957). The isolate was cryopreserved in the *Leishmania* collection of the French Reference Centre for Leishmaniasis (Montpellier, France) under the WHO code MEQU/FR/2025/LEM7200. No further genomic characterization of the isolate was performed as part of the present study.

## Results

### Case 1

A 4-year-old Quarter Horse mare was purchased in Finistère (Brittany, western France) in April 2023 and moved to Ariège (southern France) in October 2023, where she has remained since. According to the owner, the mare did not travel abroad.

During summer 2024, the horse developed a solitary ulcerated cutaneous lesion located at the base of the neck (Fig. 1). The lesion showed little clinical progression over time, and no specific clinical diagnosis was initially established.

**Figure 1:**
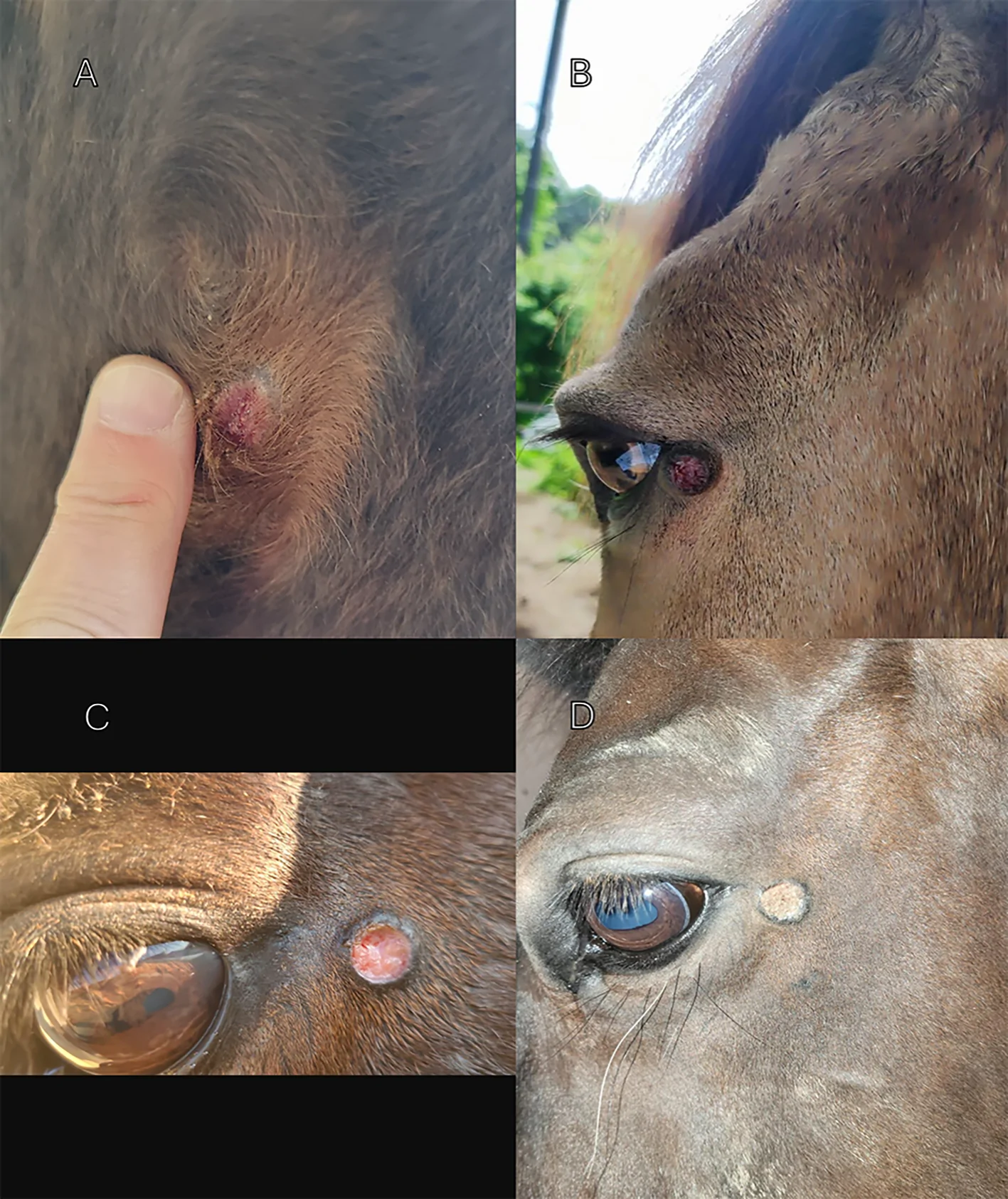
Clinical presentation of equine cutaneous lesions associated with *Leishmania martiniquensis*. (A) Solitary ulcerated lesion at the base of the neck (Case 1). (B) Periocular nodular lesion with a sarcoid-like clinical appearance (Case 2). (C) Alopecic and ulcerated periocular nodule (Case 3). (D) Same lesion after clinical resolution following intralesional meglumine antimoniate treatment (Case 3).

A skin biopsy was performed, and histopathological examination conducted in February 2025 revealed granulomatous dermatitis with numerous intracytoplasmic protozoal organisms within macrophages, morphologically consistent with *Leishmania* amastigotes. At high magnification, a nucleus and a rod-shaped kinetoplast were distinguishable in individual organisms (Fig. 2). Molecular analysis of FFPE tissue was positive for *Leishmania* spp. by PCR, and sequencing of the ITS1 locus identified *L. martiniquensis* (GenBank accession number: PX959956). Serological testing using a DAT was negative (<1:100).

**Figure 2:**
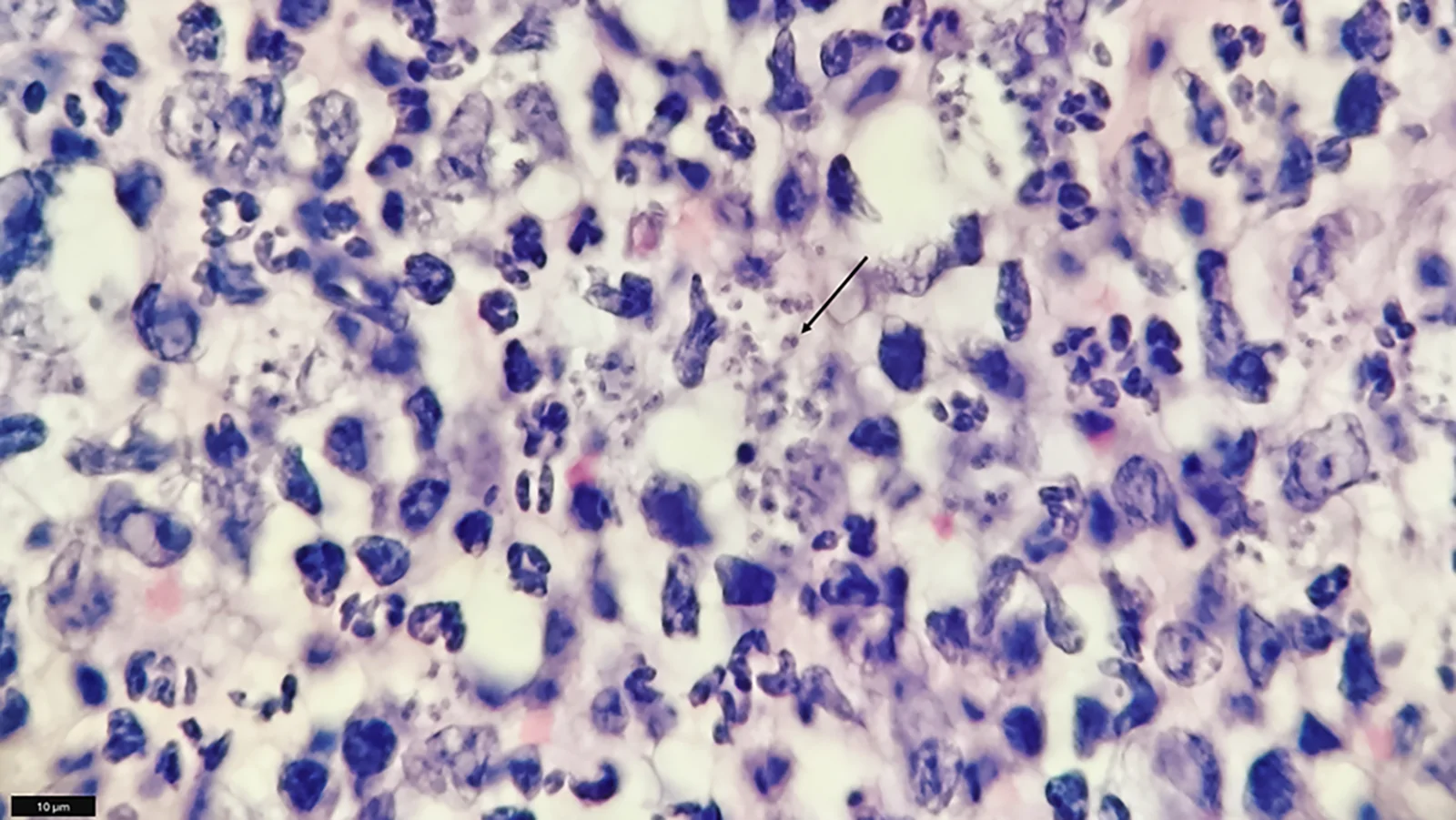
Microphotograph showing macrophages and neutrophils, with numerous intracytoplasmic protozoal amastigotes within macrophages. The amastigotes show a nucleus and a rod-shaped kinetoplast oriented approximately perpendicular to the nucleus (arrow). Hematoxylin–eosin–saffron stain; ×100 oil-immersion objective; bar = 10 μm.

No local or systemic treatment was administered. The lesion subsequently regressed spontaneously.

### Case 2

A 26-year-old mare of unspecified breed lived permanently in Dordogne (south-western France), with no reported travel history.

In December 2024, the mare developed an ulcerated nodular lesion affecting the lower eyelid, clinically compatible with a sarcoid tumor (Fig. 1). No other lesion was observed.

In June 2025, histopathological examination performed following complete surgical excision of the lesion revealed granulomatous dermatitis with numerous intrahistiocytic amastigotes. PCR analysis on paraffin-embedded tissue was positive for *Leishmania* spp., and sequencing of the ITS1 locus identified *L. martiniquensis* (GenBank accession number: PX959958). No additional medical treatment was administered. DAT serology was negative (<1:100). The lesion did not recur during follow-up (seven months after surgery).

### Case 3

A 20-year-old Dutch Warmblood (KWPN) mare first lived in Switzerland (canton of Vaud) from June 2012 to January 2020. Since January 2020, she resided in Saône-et-Loire (Burgundy, eastern France), with no reported travel since that time.

In late January 2025, the mare developed a suddenly appearing ulcerated cutaneous nodule located lateral to the left eye (Fig. 1). No specific clinical diagnosis was initially established. The lesion progressively enlarged and showed no evidence of spontaneous healing. A peripheral alopecic area surrounding the lesion was noted. No systemic signs were reported.

Histopathological examination of a skin biopsy performed in February 2025 revealed granulomatous dermatitis with numerous intrahistiocytic amastigotes. PCR analysis of FFPE tissue was positive for *Leishmania* spp., and sequencing of the ITS1 locus confirmed identification as *L. martiniquensis* (GenBank accession number: PX959957). DAT serology revealed a high antibody titer (1:6,400).

In June 2025, intralesional treatment with meglumine antimoniate was administered. In parallel, parasite isolation was successfully achieved from lesion material, and the strain was cryopreserved in the *Leishmania* collection of the French Reference Centre for Leishmaniasis (Montpellier, France) under the WHO code MEQU/FR/2025/LEM7200. Clinical follow-up showed gradual regression of the lesion, which had completely resolved by the end of November 2025, leaving an area of cicatricial alopecic.

## Discussion

The three equine cases reported here contribute to the limited body of evidence documenting cutaneous leishmaniasis caused by *Leishmania martiniquensis* in horses. Together with previous equine reports from Europe, North America, and South America [12–14], and with a bovine case reported in Switzerland [10], these observations confirm that this species or closely related agents can infect domestic ungulates across diverse geographic contexts, including outside the classical endemic areas associated with *Leishmania infantum*.

Published equine cases consistently describe a localized cutaneous form of disease with a nonspecific clinical presentation, most commonly involving lesions on the head. The French cases reported here are fully consistent with this pattern. Lesions were initially compatible with more common equine dermatological conditions, including sarcoid tumors or chronic inflammatory processes, as previously described in horses from Europe and the Americas [12–14]. This clinical overlap likely contributes to underrecognition and delayed diagnosis. Although periocular localization and sarcoid-like presentation have been repeatedly reported, histological examination did not support an association between equine leishmaniasis and sarcoids in the cases reported here.

The disease remained locally limited in the present cases, with no evidence of systemic or visceral involvement. This benign clinical course is consistent with previous equine reports of *L. martiniquensis* infection [12–14] and suggests that the clinical impact of this infection in horses is generally limited. However, the present observations do not provide information on the epidemiologic prevalence of *L. martiniquensis* in equids. In contrast, several studies conducted in endemic regions have shown that *Leishmania infantum* infection in horses and other equids is frequently asymptomatic, but is associated with relatively high seroprevalence levels [1, 3, 7, 11].

Diagnosis relied on histopathological identification of intrahistiocytic organisms morphologically consistent with *Leishmania* amastigotes, combined with molecular identification of *Leishmania* at the species level in all cases, with parasite isolation in culture achieved in one case. This approach appears to be essential, as clinical presentation alone is insufficient to suggest leishmaniasis in horses. The nucleotide sequences obtained from the three cases in France were fully identical to each other and showed 100% identity with *L. martiniquensis* sequences previously published from equine cases in Czechia and Austria [14], as well as from human cases reported in Southeast Asia, the Caribbean region, and Guyana [5, 9, 15]. They showed 99.5% identity with sequences from earlier cases reported in Germany and Switzerland [14]. This genetic homogeneity across hosts and geographically distant regions, despite limited sequence variation among some European isolates, supports stable species-level identification.

Serological responses were heterogeneous, as previously reported in equine *L. martiniquensis* infections [13, 14]. In particular, despite broadly similar localized cutaneous presentations, Case 2 was seronegative, whereas Case 3 showed a high DAT titer. The reason for this discrepancy remains unknown. Individual variability in the humoral response, differences in the timing or course of infection, and the fact that the DAT used here has not been validated for *L. martiniquensis* infection in horses may all contribute, but these possibilities remain speculative. Similar discrepancies between serological and molecular findings have also been described in equids infected with other *Leishmania* species, including *Leishmania braziliensis*, further supporting the limited diagnostic value of serology alone in this host [18].

From an epidemiologic perspective, the three French cases were geographically dispersed and not temporally clustered, supporting a sporadic pattern of detection rather than active localized transmission. In two cases, long-term residence in France without recent travel was compatible with local acquisition, although this cannot be formally established. For Case 3, previous residence in Switzerland and the unknown interval between infection and clinical expression preclude any conclusion regarding the country of acquisition. No conclusion can be drawn regarding transmission routes or reservoirs.

Hypotheses concerning vectors or reservoirs of species belonging to the subgenus *Mundinia* remain speculative and are not supported by epidemiologic data in horses [3, 12, 14]. The rarity of reported cases and the absence of structured field studies currently preclude any firm epidemiologic interpretation, underlining the importance of continued descriptive reporting.

The transmission of *L. martiniquensis* remains poorly understood. Biting midges of the genus *Culicoides* have been demonstrated to support transmission of *Mundinia* parasites under experimental conditions [2], and natural infection with *L. martiniquensis* has been reported in *Culicoides peregrinus* in Thailand [8]. Although *Phlebotomus perniciosus* is present in France [16], experimental studies using two strains of *L. martiniquensis*, including an isolate from a European horse, showed that this sand fly species did not support mature parasite development and was therefore not considered a competent vector for this species [17]. However, these observations do not identify the vector or vectors involved in the French context. In particular, observations involving *Culicoides peregrinus* in Thailand cannot be directly extrapolated to *Culicoides* fauna in France, and no entomological investigation was conducted around the cases reported here.
